# Pleomorphic Parotid Adenoma in a Child Affected with Cri du Chat Syndrome: Clinical, Cytogenetic, and Molecular Analysis

**DOI:** 10.3390/ijms251910664

**Published:** 2024-10-03

**Authors:** Cesare Danesino, Federico Biglioli, Laura Moneghini, Roberto Valli, Carla Olivieri, Barbara Testa, Chiara Baldo, Michela Malacarne, Andrea Guala

**Affiliations:** 1 Department of Molecular Medicine, University of Pavia, 27100 Pavia, Italy; carla.olivieri@unipv.it; 2 Maxillo-Facial Surgery Unit, Health Sciences Department, University of Milan, 20122 Milan, Italy; federico.biglioli@unimi.it; 3 Unit of Pathology, San Paolo Hospital, 20122 Milan, Italy; laura.moneghini@asst-santipaolocarlo.it; 4 Department of Medicine and Surgery, University of Insubria, 21100 Varese, Italy; roberto.valli@uninsubria.it; 5 UOC Laboratorio di Genetica Umana, IRCCS G. Gaslini, 16147 Genova, Italy; barbaratesta@gaslini.org (B.T.); chiarabaldo@gaslini.org (C.B.); michelamalacarne@gaslini.org (M.M.); 6 Pediatric Unit, Castelli Hospital, 28921 Verbania, Italy; gualaandrea0@gmail.com

**Keywords:** parotid pleomorphic adenoma, Cri du Chat syndrome, array CGH, molecular analysis

## Abstract

Salivary gland pleomorphic adenoma (SGPA) is the most common type of benign epithelial tumor; it is observed more commonly in females (with a female-to-male ratio of 1.43:1), and the age at diagnosis ranges between 40 and 59 years, with only 2% of cases diagnosed before age 18. Cri du Chat (CdC) is a rare syndrome caused by deletions of various sizes in the short arm of chromosome 5. Tumors in CdC patients are extremely rare: in Danish, Spanish, Australian, and Japanese groups of cases, no tumors have been reported, while a few cases have been described among 321 CdC patients collected in Italy and Germany. These cases all involve tumors that appear at a young age. We here report the case of a parotid pleomorphic adenoma in an 8-year-old boy with CdC. Exome analysis did not identify variants certainly significant for the development of SGPA. A CGH array, analyzed both in peripheral blood and tumor samples, failed to recognize anomalies previously associated with SGPA but identified a de novo duplication in 7p15.2, which contains part of a gene, *SKAP2*, in which the increased copy number is associated with the development of a different type of tumor such as pancreatic duct adenocarcinoma. The assumption that the duplication in 7p15.2 is relevant for the development of SGPA in our patient implies that CGH array studies must be included early in life in routine work-ups of CdC to identify CNVs with possible pathogenic roles for tumor development. This is particularly also relevant in relation to the severely impaired possibility for patients with CdC to report discomfort or pain related to tumor development. Constitutional CNVs in addition to the deletion in 5p should also be extensively studied to verify if their presence in some patients could explain why, in these cases, tumors develop at an age younger than expected.

## 1. Introduction

Salivary gland pleomorphic adenoma (SGPA) is the most common type of benign epithelial tumor and accounts for at least 70% of them. SGPAs are observed more commonly in females (with a female-to-male ratio of 1.43:1), with an age at diagnosis between 40 and 59 years, and only 2% of cases diagnosed before age 18 [1]. Its rarity in young ages is also confirmed in studies collecting large series of cases [2,3].

Cri du Chat (CdC) is a rare syndrome caused by deletions of various sizes in the short arm of chromosome 5. Tumors in CdC patients are extremely rare; for instance, in the reports about Danish [4] and Spanish [5] groups of CdC (62 and 70 patients, respectively), no type of tumor was reported; similarly in unpublished CdC patients from Australia and New Zealand [6] and Japan [7] (102 and 111 cases, respectively), as far as we are aware, no cases of tumors were observed.

Our group reported that in 321 CdC patients collected in Italy and Germany, four patients were affected by various types of tumors (three malignant tumors and one benign tumor). In the three patients affected by cancer (thyroid papillary carcinoma, gastric carcinoid, and breast cancer), the age of onset of each cancer was much earlier than expected for its natural history in the general population [8]. While no new cases have been added since to the German registry [9], a new CdC patient with cancer, with early-onset (29 yrs old) oesophageal carcinoma, was described in Italy [10].

With the aim to collect and share all available information about tumors, both benign and malignant, in CdC patients, we here report the case of an SGPA in an 8-year-old boy with CdC.

### Case Report

The patient, a male, the first child of healthy unrelated parents (father’s age, 45, and mother’s age, 36), was born in 2014 after an uneventful pregnancy, with cesarean section because of podalic presentation; the child’s birth weight was 2710 g (25 °C), length 44 cm (5 °C), and head circumference 32.5 cm (10 °C) (centiles as published for CdC patients) [11].

At birth, the presence of dysmorphic features such as a round face, microcephaly, an epicanthal fold, low set ears, micrognathia, a simian crease associated with severe axial hypotonia, and a cat cry suggested clinically the diagnosis of Cri du Chat Syndrome, which was shortly confirmed by routine cytogenetic analysis: 46,XY,(del5)(p15.33–p13.3). At 1 month, an EEG showed a normal pattern.

No major malformations were observed, but severe developmental delay was clear shortly after birth.

When the patient was three and a half year is weight was 10.900 kg (25 °C), the length was 94 cm (50 °C) and head circumference was 43.5 cm (25 °C) [11]. He showed global hypotonia and was unable to walk, able to sit without support, and moved by shuffling. At this age, he was not able to say any words. Follow-up demonstrated only minor motor improvements (in May 2024, he was still not able to walk but was able to stand with support); speech development was severely delayed and limited to a few sounds. It was not possible to administer common tests to assess development scores.

At age 8, a swelling of the left sub auricular region was further indagated by ultrasound, which demonstrated in the left parotid gland the presence of a hypoechogenic ovalar formation, which was not vascularized in which the size was 16 × 11 mm. A needle biopsy demonstrated the presence of a pleomorphic adenoma.

The patient at the time of the surgery in March 2023 was 116 cm in height and 20 kg in weight, both at the 5th centile for CdC patients [11].

**Figure 1:**
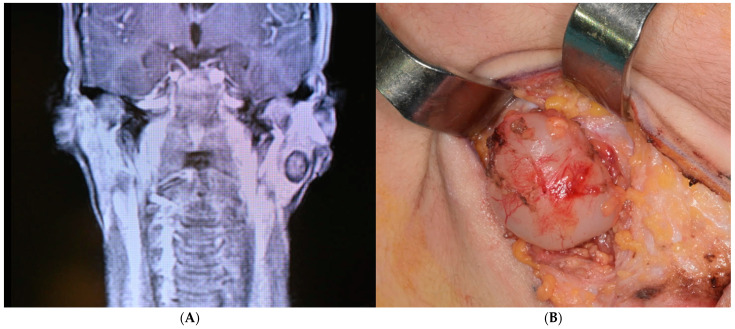
(**A**): MRI image of the tumor. (**B**): appearance of the lesion at surgery.

Surgery followed our surgical step for SGPA: a face-lift type of incision allowed us to lift a skin flap just in front of the ear and expose the superficial aspect of the parotid gland. An extracapsular dissection allowed us to maintain the gland integrity and to identify just a couple of facial nerve branches, which were left untouched and checked by electrostimulation. The surgical sample consisted of a neoformation measuring 2.8 g and 2 × 1.8 × 1.3 cm, greyish-white, and homogeneous when cut. No complications occurred after surgery; facial nerve function was perfectly conserved, and the scar was well hidden. No recurrence was evident 12 months after surgery, neither clinically nor after ultrasound examination. MRI and intra-operatory images are reported in Figure 1A,B. 

## 2. Material and Methods

### 2.1. Array CGH

Array CGH was performed in 2023 on both peripheral blood and a sample from the adenoma of the patient and peripheral blood from the parents using routine methods.

### 2.2. Molecular Investigation

The patient was included in a group of CdC cases in whom WES analysis was performed in the trio for a project aiming to identify genetic variants in addition to the deletion, which could explain specific clinical signs not usually reported in Cri du Chat Syndrome. DNA from peripheral blood and parotid tissue was extracted using the phenol/chloroform method; dried DNA pellets were stored at −20 until the time of molecular analyses.

Genomic DNA was used for preparing whole-exome sequencing libraries (1.5 µg from peripheral whole blood) with a commercial kit (Agilent SureSelectXT HumanAllExon V5+UTRs; Agilent Technologies, Santa Clara, CA, USA) and sequenced on a HiSeq1000 platform (paired-end 2 × 100 nt; Illumina, San Diego, CA, USA).

Reads were quality-filtered and aligned to the reference human genome sequence (GRCh37/hg19) with an ISAAC aligner [12]. VarSeq v1.4.5 (Golden Helix, Inc., Bozeman, MT, USA) was used for genomic variant annotation, and only variants with a minimum quality score of 20, a minimum read depth of 30X, and a population frequency of <5% (gnomAD) were selected; the effect of selected variants on protein function (damaging or tolerated) was reported in SIFT and Polyphen.

The results were filtered for rare variants under models of autosomal recessive inheritance (homozygous or compound heterozygous) and de novo variants; the results are summarized in Table 1.

We confirmed the relevant variants by Sanger sequencing. The primers used for the amplification and sequencing were designed using Primer3web (https://primer3.ut.ee/; accessed 1 October 2022). PCRs were performed in 25 μL of reaction volume containing 50 ng of genomic DNA, 0.5 μm primers, and a 2 μL dNTP mixture (2.5 mm each) using routine methods.

## 3. Results

### 3.1. Array CGH

**Figure 2:**
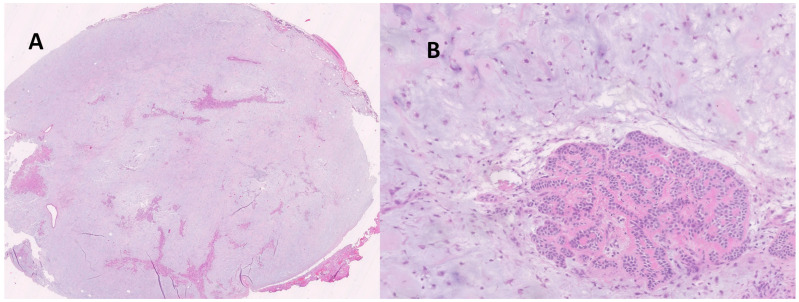
(**A**): at low magnification, it is evident that the neoformation is well defined compared to the surrounding glandular tissue (hematoxylin/eosin). (**B**): at higher magnification, the characteristic chondromyxoid stroma is evident. At the bottom right, there is also an epithelial aggregate, as typically appears in pleomorphic adenoma (hematoxylin/eosin).

The results of array CGH on both peripheral blood and tumoral tissue, in addition to the known deletion on 5p-, disclosed a large deletion in 8q limited to the adenoma. An amplification on chromosome 7 p15.2 involving *SKAP2* and two small deletions on chromosomes 10 and 14 were present in both tissues; see Table 1 and Figure 2 for details. All the anomalies were de novo, and the parents did not show any anomalies in the CGH array.

### 3.2. Molecular Investigation

Table 2 contains the results of exome analysis and lists the rare variants filtered under models of autosomal recessive inheritance (homozygous or compound heterozygous) and de novo variants.

### 3.3. Pathology

At histological examination, the specimens showed typical morphology of pleomorphic adenoma of the salivary glands: well-defined proliferation (Figure 2A) with a prevalence of chondromyxoid stroma with a poor epithelial component consisting of aggregation of bilayered ducts without atypia and mitotic activity (Figure 2B). In the context of the adenoma, there were histiocytic aggregates and foci of squamous metaplasia compatible with the performance of a previous fine needle aspiration. The lesion is surrounded by a thin rim of glandular and connective tissue. The observed morphology confirmed the diagnosis of pleomorphic adenoma already suggested after cytological examination.

### 3.4. Cell Colture and Cytogenetics

Chromosome analysis on blood lymphocytes confirmed the deletion of the short arm of chromosome 5 in the patient, 46,XY,(del5)(p15.33–p13.3), and showed normal male and female karyotype in the parents. Cell cultures from the tumor sample allowed the growth of only a few cells, which was not suitable for cytogenetic or molecular studies.

## 4. Discussion

Pleomorphic adenoma of the parotid gland is a common benign tumor of adult age [1] and, to the best of our knowledge, was never reported in association with Cri du Cat Syndrome. Reports of the development of various types of tumors in CdC patients are limited to the papers by Guala et al. [8] and Danesino et al. [10], while collections of clinical data from a series of patients or personal communication from some family associations failed to observe any benign or malignant neoplasms in CdC syndrome [4,5,6,7,9].

A possible explanation for this uneven reporting of tumor cases among CdC patients from various countries, with a peculiarly high number of cases from Italy, might be related to patients’ age. In fact, the mean age of the group of Italian CdC from which the data are collected is higher than the mean age of other groups of cases reported [4,5,6,7]. In addition, A.B.C., the Italian charity for CdC syndrome, is very active in updating all clinical data in their annual meeting, and the intensive and long-lasting rehabilitation program ongoing among A.B.C. associates helps to collect a large set of data.

### 4.1. Clinics

Clinical presentation limited to swelling was similar to other cases of SGPA; surgery did not differ from our routine SGPA removal procedure, and intra-operatory findings were as expected. Follow-up is the same as in adult cases, consisting of annual ultrasound controls and outpatient clinical examinations scheduled for ten years.

### 4.2. Pathology

Light microscopy of the sample confirmed a typical SGPA.

### 4.3. Cytogenetics and Molecular Investigations

Several papers have discussed routine and molecular cytogenetics of SGPA, also demonstrating the presence of somatic chromosomal abnormalities in very old studies [13].

In most cases, no constitutional abnormality was observed, but in tumor samples, the extensive involvement of chromosome 8q was reported by Sandros et al. [14]. Recurrent translocations, t(3;8)(p21;q12), and t(5;8)(p13;q12), allowed for the identification of several genes involved in SGPA oncogenesis, namely *PLAG1*, *CTNNB1*, and *LIFR*, mapped, respectively, in 8q12.1, 3p22.1, and 5p13.1 [15,16]. The molecular pathogenic mechanism relates to the aberrant expression of *PLAG1* when driven under the control of promoters of the distinct translocation partner genes; it was reviewed by Stenman et al. 2022 [17] and also involves *IGF2*. Our patient does not carry any variant of *IGF2.*

**Figure 3:**
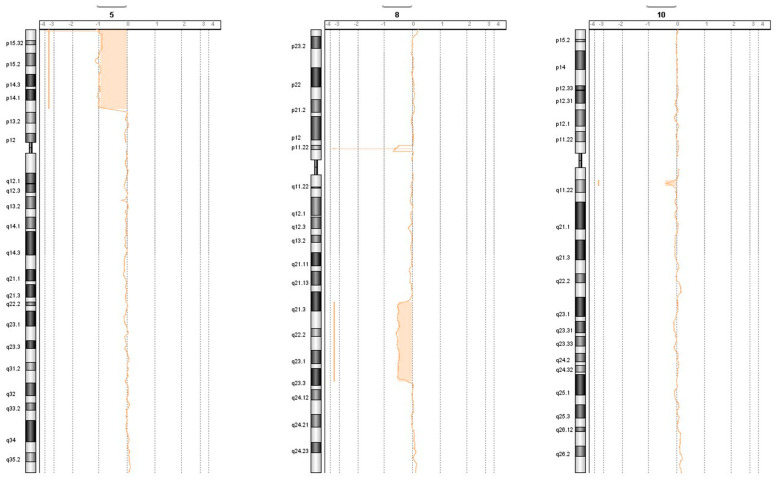
Results of the array on peripheral blood and SGPA: the deletions on chromosomes 5 (constitutional), 8 (somatic), and 10 (constitutional) are depicted.

In our case, the deletion observed in the tumoral tissue does not include band 8q12.1; the *PLAG1* gene is not involved in any structural rearrangement (Figure 3 and Table 1) and does not contain any variant. *CTNNB1* does not contain any variant, and band 3p22.1 is not involved in any rearrangement. As for *LIFR*, it is not included in the constitutionally deleted 5p region, and our patient carries only the variant 5-38528865 (C>T), which was interpreted as benign.

Table 1 lists the regions demonstrated to be quantitatively altered by CGH array and the genes included in these regions. 

Overall, the regions found to be deleted or duplicated in our patient by array CGH did not overlap with those reported in previous studies on SGPA [18,19] and in a recent study on single cells from SGPA [20]. The observation of the involvement of multiple chromosomal regions suggests that the etiology of SGPA results from many independent genomic alterations [20].

### 4.4. CGH Array

#### 4.4.1. Chromosome 5, Deletion p15.33–p13.3

SGPA was never reported in association with CdC, so the genes located in 5p- deletion are unlikely to have relevance in the development of the tumor; among them, *TERT* (5p15.33) is related to tumor development, but it is not expressed in a series of SGPA and normal parotid tissue [21], and its involvement in salivary gland was only reported in a case of a myoepithelial carcinoma gland in a 76-year-old lady [22]. *AHRR* (see Table 1 and Table 2) carries a likely benign missense variant, but the expression of the gene was reported to be extremely low (not quantifiable) in healthy parotid glands as well as in pleomorphic adenomas [23]. CDH10 (see Table 1 and Table 2) contains an intronic variant.

#### 4.4.2. Chromosome 7, Duplication p15.2

The duplication of region 7p15.2 is present both on the peripheral blood (PB) and tumor sample but absent in the parents; the duplication involves exons 1–4 of the *SKAP2* gene and is not included among the known copy number variations (CNVs).

An overexpression of this gene, in most cases due to increased copy number, was reported to be likely associated with the development of pancreatic duct adenocarcinoma [24]; so, in our case, the de novo increase in copy number variation of a region containing part of this gene suggests a possible relevance in SGPA development.

A similar deleterious effect associated with the tumor development of an only partially duplicated gene, like that found in SKAP2, was demonstrated in *RUNX1* by Marletta et al. [25].

#### 4.4.3. Chromosome 8, Deletion 21.3–23.3

In addition to previous comments on the chromosome 8 region typically deleted in SGPA, the genes included in the somatic deletion in our patient were not reported in the medical literature in association with SGPA (check in OMIM/UCSC/PUBMED using the words “salivary gland/adenoma/parotid/Cri du Chat/5p-” in all possible combinations failed to retrieve relevant papers, updated May 2024).

#### 4.4.4. Chromosome 10, Deletion q11.22

The deletion in 10q11.22 is present in both PB and SGPA; it is de novo and contains several genes, none of which are related to SGPA (check in OMIM/UCSC/PUBMED using the words “salivary gland/adenoma/parotid/Cri du Chat/5p-” in all possible combinations failed to retrieve relevant papers, updated May 2024).

#### 4.4.5. Chromosome 14, Deletion q21.3

This deletion, observed in both PB and tumoral tissue, is de novo, not present in the database of genomic variation, and contains the gene *MDGA2* (Table 1), which is not expressed in salivary glands and was never discussed in relation to CdC. The patient does not carry any variant of this gene.

### 4.5. WES

Table 2 contains the rare variants found after exome analysis in the patient and filters models of autosomal recessive inheritance (homozygous or compound heterozygous) and de novo variants. Among the genes listed, (i) *AHRR* and *CDH10 are* included in the deleted region; see comments in “chromosome 5 deletion”. (ii) *MAGI3, TTC38,* and *G2E3* are not related to salivary gland development or SGPA, contain likely pathogenic variants, but no associated clinical phenotypes are reported in OMIM. (iii) *MAPK1* contains an in-frame deletion; Kolary-Siekierska et al. [26] reported that this gene inhibits apoptosis and autophagy and a decreased gene expression in SGPA can be observed; they also suggested that apoptosis and autophagy may be of some relevance for the development of SGPA.

## 5. Conclusions

The observation of an SGPA in a young patient is uncommon [27], and in general, reports of tumors, benign or malignant, in CdC patients are extremely rare [9,10]. In our patient, we did not find evidence using exome analysis of any single gene variant that could act clearly as an oncogenic predisposing factor.

Our CGH array disclosed a duplication in 7p15.2, both in the PB and tumor sample, which contains part of a gene, *SKAP2*, whose increased copy number is associated with the development of a different type of cancer [24].

CNVs are an important component of genetic variation, with a possible role in susceptibility to many types of diseases, including cancer, although their role as risk factors for tumors is not yet fully understood [28].

The assumption that the duplication in 7p15.2 is relevant for the development of SGPA in our patient implies that a routinely available genetic test such as CGH array studies, must be included in routine work-up of CdC to identify CNVs with possible pathogenic roles for tumor development [28]. Finding genetic variations in addition to the 5p- deletion causing CdC will help to better care for patients when these variations behave as risk factors for different complications, including tumors. Moreover, they might explain, in some cases, the uncommon clinical aspects of a patient’s phenotype.

CGH arrays are currently a cheap test often routinely performed in place of conventional cytogenetic analysis. It follows that adding a CGH array into the routine genetic analysis of a patient clinically diagnosed as affected by CdC syndrome, will not really impact the cost/benefit ratio for obtaining the genetic diagnosis. The latter, when obtained by CGH array, will be much more rich in information useful to fully understand genotype–phenotype correlations.

This is particularly relevant in relation to the severely impaired possibility for CdC patients to report discomfort or pain related to tumor development [10].

As a working hypothesis, constitutional CNVs in addition to the deletion in 5p should be extensively studied to verify if their presence in some patients could also explain why, in these cases, tumors develop at an age younger than expected (9–10).

## Figures and Tables

**Table 1:** List of deletions in blood and SGPA with the genes included; an amplification on chromosome 7 was also observed.

Chr	Cytoband	Start	Stop	#Probes	Amplification	Deletion	Gene Names (Partial List)	Notes
chr5	p15.33–p13.3	22149	32688654	2140	0	−0.94386	PLEKHG4B, LRRC14B, CCDC127, SDHA, PCDG6, AHRR, LOC100301782, SOF55, EXOC3, LOC25845, SLC9A3, CEP72, TPPP, ZDHHC11, BRD9, TRIP13, NKD2, SLC12A7, SLC6A19, SLC6A18, TERT, CLPTM1L, SLC6A3, LPCAT1, SDHAP1, LOC728613, MRPL36, NDUFS, IK2A, IRX2, C5orf83, IRX1, LOC340094, ADAMTS16, MCM9, FLJ33360, MED10, UBE2QL1, LOC55167, NSUN2, SRD5A1, PAPD7, ADYC2, SOF249, FASTK, TMRC, SEMASA, SNORD123, TAS2R1, LOC285692, FAM173B, CCT5, CHML, MARCHE, ROH1, ZNRD3B, DAPT, CTNND2, DNAH5, TRIO, FAM105A, FAM105B, ANKH, FBLX7, MARCH11, ZNF622, FAM134B, MYO10, LOC285696, BASP1, CHD18, LOC728411, CDH12, PMCH1L, PRDM9, CDH10, CDH9, DNHD6, RANSE, C5orf22, PDDZ, GOLPH3, MTMR12, ZFR, SUB1	Present also in blood sample. Related to Cri-du-chat Synd
chr7	p15.2	26888579	26916319	5	0.89805	0	SKAP2	Amplification present also in blood sample. Not present in Database of Genomic Variants.
chr8	q21.3–q23.3	90138085	116409381	2001	0	−0.499885	RIPK2, OSGIN2, NBN, DECR1, CALB1, TMEM64, NECAB1, TMEM55A, OTUD6B, LRRC59, SLC26A7, RUNX1T1, C8orf83, FAM92A1, RBM12B, SOF389, TMEM67, PDP1, CDH17, GEM, RAD54B, KIAA1492, ESRP1, PGR19L4, INTS8, CCNE2, TP53INP1, C8orf83, PLEKHF2, C8orf34, GDF6, UBAC2, MTREFD1, DPT, PTPSD3, SDCC, PCPG, FSOF5, TFSY5, MTDH, LAPTM4B, MATN2, RIPLO, SONORA72, C8orf47, HRSRIP, POP1, NIPLA2, KCN2, STK3, OSR2, YSP318, MIRS99, MIRS75, C00XC, RGS22, FBXO43, POLR2K, SPAG1, RNF19A, ANK846, SNX31, PAPBC1, WHAC, ZNF706, NACAP1, GHRL2, NALCD, RRM2B, UBRS, ODF1, KLF10, AZIN1, ATP6V1, C8orf56, LABP, ZC6, CTHRC1, SLC25A2, DCAF13, RIMS2, TM75A4, DPYS, LRIP12, F2PM2, OXRI, ABRA, ANPG11, PSF02, EFR13C, TS6S, TMEM74, TRHR, NUCD81, ENY2, PKHD11A, EBA9, GOLYSN, KCNV1, C5MD3, MIR2053	Deletion present ONLY in tumor sample. Estimated percentage of cells: 57%.
chr10	q11.22	46158156	48115525	43	0	−0.40818	ANUBL1, FAM21C, AGAP4, PTPN20B, PTPN20A, BMS1P5, BMS1P1, FAM35B, STY15, GPRIN2, PYRY1, LOC728643, ANXA8, ANXA8L1, FAM25B, FAM25C, FAM25G, LOC64826, FAM35B2, ANTXRL, ANXA8L2, FAM21B	Deletion present also in blood sample. Only partially reported in Database of Genomic Variants.
chr14	q21.3	47937337	47974893	6	0	−0.977033	MDGA2	Deletion present also in blood sample. Not present in Database of Genomic Variants.

**Table 2:** Rare variants found in exome analysis in the patient and filtered under models of autosomal recessive inheritance (homozygous or compound heterozygous) and de novo variants. *: included in the deleted region; ^: likely pathogenic.

Genes	Genotype	Mutation Type
*CHRNA3*;	Autosomal recessive	in frame deletion
*ZNF254*; AHRR *;		missense
*CACNA1B*;		splice variant
*USP9X*; *TEX11*;	X-linked recessive	splice variant
*PLP2*; *VSIG4*; *RTL4*;		missense
*OR10V1*; *RBM19*; *TMEM132C*; *TTN*; *PLEC*;	Compound Heterozygote	missense
***MAGI3 ^***;	de novo	missense/splice variant
*FRAT2*; *ACSS3*; *C12orf49*; *TTLL13P*; *PDP2*; *TTC38 ^*; *SPTBN4*; *MROH5*; *GRIPAP1*; *TF3*; *DOCK11*;	de novo	1 missense
*TRMT1L*; *CFAP46*; *BRAP*; *LLGL2*; CDH10 *; *BRD8*;	de novo	splice variant
*KDM2B*; *RABEP2*; *SLC8A2*; *POU3F3*; *MAPK1*; *CRHBP*; *CNKSR2*; *LRCH2*;	de novo	in frame deletion
*CSMD2*; ***G2E3 ^***; *WIZ*; *NR1H2*; *STAB1*; *KLF8*;	de novo	frameshift

## Data Availability

WES general data are available on request.
